# The preparation of new functionalized [2.2]paracyclophane derivatives with *N*-containing functional groups

**DOI:** 10.3762/bjoc.11.50

**Published:** 2015-04-07

**Authors:** Henning Hopf, Swaminathan Vijay Narayanan, Peter G Jones

**Affiliations:** 1 Institut für Organische Chemie, Technische Universität Braunschweig, Hagenring 30, D-38106 Braunschweig, Germany, Fax: (+49)531-391-5388; 2 Institut für Anorganische und Analytische Chemie, Technische Universität Braunschweig, Postfach 3329, D-38106 Braunschweig, Germany, Fax: (+49)531-391-5387

**Keywords:** azides, crownophanes, cyclophanes, isocyanates, stereochemistry, X-ray analysis

## Abstract

The two isomeric bis(isocyanates) 4,12- and 4,16-di-isocyanato[2.2]paracyclophane, **16** and **28**, have been prepared from their corresponding diacids by simple routes. The two isomers are versatile intermediates for the preparation of various cyclophanes bearing substituents with nitrogen-containing functional groups, e.g., the pseudo-*ortho* diamine **8**, the bis secondary amine **23**, and the crownophanes **18** and **19**. Several of these new cyclophane derivatives (**18**, **19**, **22**, **26**, **28**) have been characterized by X-ray structural analysis.

## Introduction

**Figure 1:**
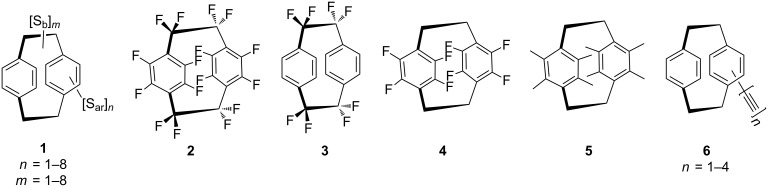
A selection of highly substituted/functionalized [2.2]paracyclophanes.

Although hundreds of mono- und disubstituted derivatives of [2.2]paracyclophane [3–4] have been described since its initial preparation [5], relatively little is known about more highly substituted and highly functionalized [2.2]paracyclophanes, which are displayed in general form in formula **1** in Figure 1.

In this general structure we distinguish between bridge substituents [S_b_]*_m_*_,_ where the number of substituents *m* can lie between 1 and 8, and substituents that are anchored in the benzene rings, [S_ar_]*_n_*, *n* again ranging from 1 to 8. Of course, these two types of substituents will display different reactivities since they are bonded to differently hybridized carbon atoms. In a way, the rigid cyclophane carbon framework may be regarded as a hexadecavalent “superatom” that can bind up to 16 substituents in a geometrically clearly defined way. If both bridges and rings are fully substituted (**1**, *m* = 8, *n* = 8) we are dealing with an elongated, American football-type of carbon core to which 16 substituents are bound in a pincushion-like fashion. If only 8 substituents are anchored on one side of the cyclophane molecule, all pointing in the same direction, a structure results that bears functional groups on the one half of the molecule whereas the other consists of aliphatic and aromatic C–H bonds only. It is very likely that these geometries will have a strong influence on chemical and physical properties (e.g., solubility behavior) of the respective derivatives. Clearly, many other arrangements of important functional groups are possible, depending on the number of substituents (S_b_ and S_ar_) and their locations. So far the most thoroughly studied, more than singly substituted system is the pseudo-*geminally* disubstituted derivative in which the two (identical or non-identical) substituents are directly above/below each other (see below).

To the best of our knowledge the first fully functionalized [2.2]paracyclophane was the fluorocarbon perfluoro[2.2]paracyclophane (**2**) first prepared by Dolbier and co-workers [6]. The corresponding perchloro and perbromo derivatives are apparently unknown.

Other notable oligofluoro hydrocarbons are the octafluoro derivative **3** with eight fluorine substituents in S_b_ positions [7–10], and its isomer **4** with the eight fluorine substituents attached to the two benzene rings [11–12].

These paracyclophane derivatives are of interest as substrates for the preparation of highly and/or perfluorinated parylene derivatives, the polymer obtained by flash vacuum pyrolysis of the respective paracyclophane precursors. Among the highly alkyl-substituted [2.2]paracyclophanes the octamethyl compound **5** is the most highly substituted derivative presently known [13–14].

Finally, various ethynylated derivatives **6** have been described by us recently [15], compounds that, inter alia, are of interest in materials science [16–18].

**Figure 2:**
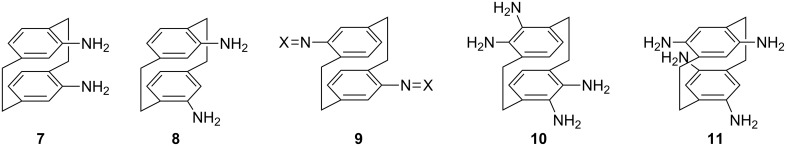
A selection of [2.2]paracyclophanes carrying several nitrogen-containing substituents.

Among the oligo- or polysubstituted paracyclophanes with preparative potential, we think that those bearing nitrogen-containing functional groups, although known for many years [19], deserve more attention. A case in point is the (achiral) pseudo-*geminal* diamine **7** (Figure 2) [20], which has been used as a reusable spacer for “topochemical reaction control in solution” [21].

We are interested in generalizing the methods described for the preparation of **7** and in the present contribution to concentrate on the preparation of the pseudo-*ortho*-diamine **8**, which is not only chiral but also offers numerous possibilities for further transformations. Related to the investigation of **8** are compounds of type **9**, in which the *N*-carrying functional groups are now attached to the pseudo-*para* positions. With identical atoms/groups X these compounds are of course achiral because of their inversion symmetry. A specific example of this type of derivative would be that with X = CO, i.e., the bis(isocyanate) (see below).

Once we have developed high-yielding routes to **8** and **9**, we plan to apply them to the preparation of the isomeric tetraamines **10** and **11** from the corresponding, already available tetraacids [22–23].

## Results and Discussion

### Preparation and reactivity of 4,12-diamino[2.2]paracyclophane (**8**, pseudo-*ortho-*diamine **8**)

**Scheme 1:**
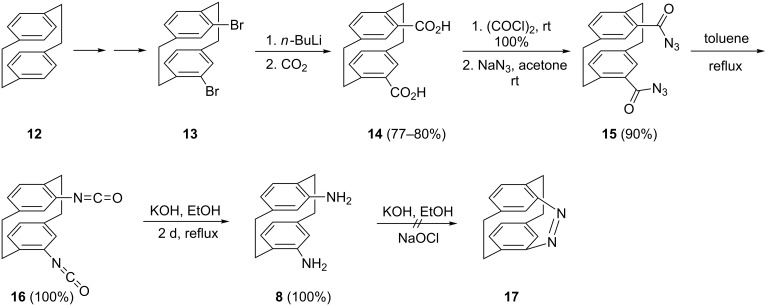
The preparation of 4,12-diamino[2.2]paracyclophane (**8**).

The diamine **8** was obtained by the sequence summarized in Scheme 1.

The crucial intermediate in this route is the pseudo-*ortho-*diacid **14**, which has been prepared several times before [24–27] by various routes.

We prefer to start with the parent hydrocarbon [2.2]paracyclophane (**12**), convert this to the dibromide **13**, metalate it to the corresponding dilithio derivative, from which **14** is finally obtained by a CO_2_ quench [25–26]. On treatment of **14** with oxalyl chloride in anhydrous dichloromethane in the presence of catalytic amounts of DMF in dichloromethane (10% solution) at room temperature [27], the expected bis(acyl chloride) was obtained in quantitative yield and characterized by the usual spectroscopic methods (see Supporting Information File 1). Subjecting this intermediate to sodium azide treatment in acetone at room temperature provided the isolable azidocarbonyl derivative **15** in 90% yield; for its analytical data see Supporting Information File 1. Compound **15** can even be purified by silica gel column chromatography although it was noted that Curtius rearrangement to the bis(isocyanate) **16** slowly set in. Hence **15** was employed in the Curtius step (toluene, reflux) without further purification and at about 95 °C the evolution of nitrogen gas could be clearly seen. The bis(isocyanate) **16** was isolated in quantitative yield and characterized by its spectroscopic and analytical data (see Supporting Information File 1). Finally, when **16** was first refluxed in ethanol and the resulting solution (presumably containing the corresponding bis(urethane) is subsequently treated with an aqueous solution of potassium hydroxide (20%), 4,12-diamino[2.2]paracyclophane (**8**) was obtained in quantitative yield. Since we had converted its pseudo-*geminal* isomer **7** by sodium hypochlorite oxidation into the corresponding azo compound [20] we also attempted this transformation for **8**.

The intended, chiral azo compound **17** was, however, not produced. Presumably the two amino functions are too far apart for intramolecular bridging and the strain of the desired **17** would be too high for a successful ring closure.

**Scheme 2:**
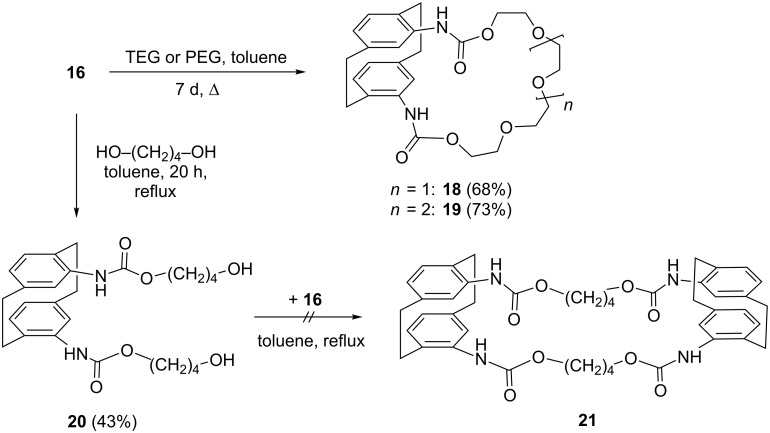
Preparation of cyclic and acyclic urethanes from 4,12-diisocyanato[2.2]paracyclophane (**16**).

Reacting **16** with tetraethylene glycol (TEG) under high dilution conditions (toluene, reflux, 7 d) provides the crownophane **18** in 68% yield (Scheme 2). Likewise, replacement of TEG by pentaethylene glycol (PEG) resulted in the formation of the next higher homolog **19** (73%). Both compounds were readily characterized by their analytical data (see Supporting Information File 1) and also by an X-ray structural analysis of **18**.

Several features are common to all structures presented in this paper. The usual features of cyclophane strain include the following. The bridge C–C single bonds are lengthened to 1.58–1.60 Å, the bridge sp^3^-bond angles are widened to ca. 112°, and the bridgehead angles within the six-membered rings are narrowed to 116–118°. These rings display a flattened boat conformation (deviations of bridgehead atoms 0.14–0.17 Å) and are mutually parallel (interplanar angles ≤ 1°, calculated without the bridgehead carbons).

**Figure 3:**
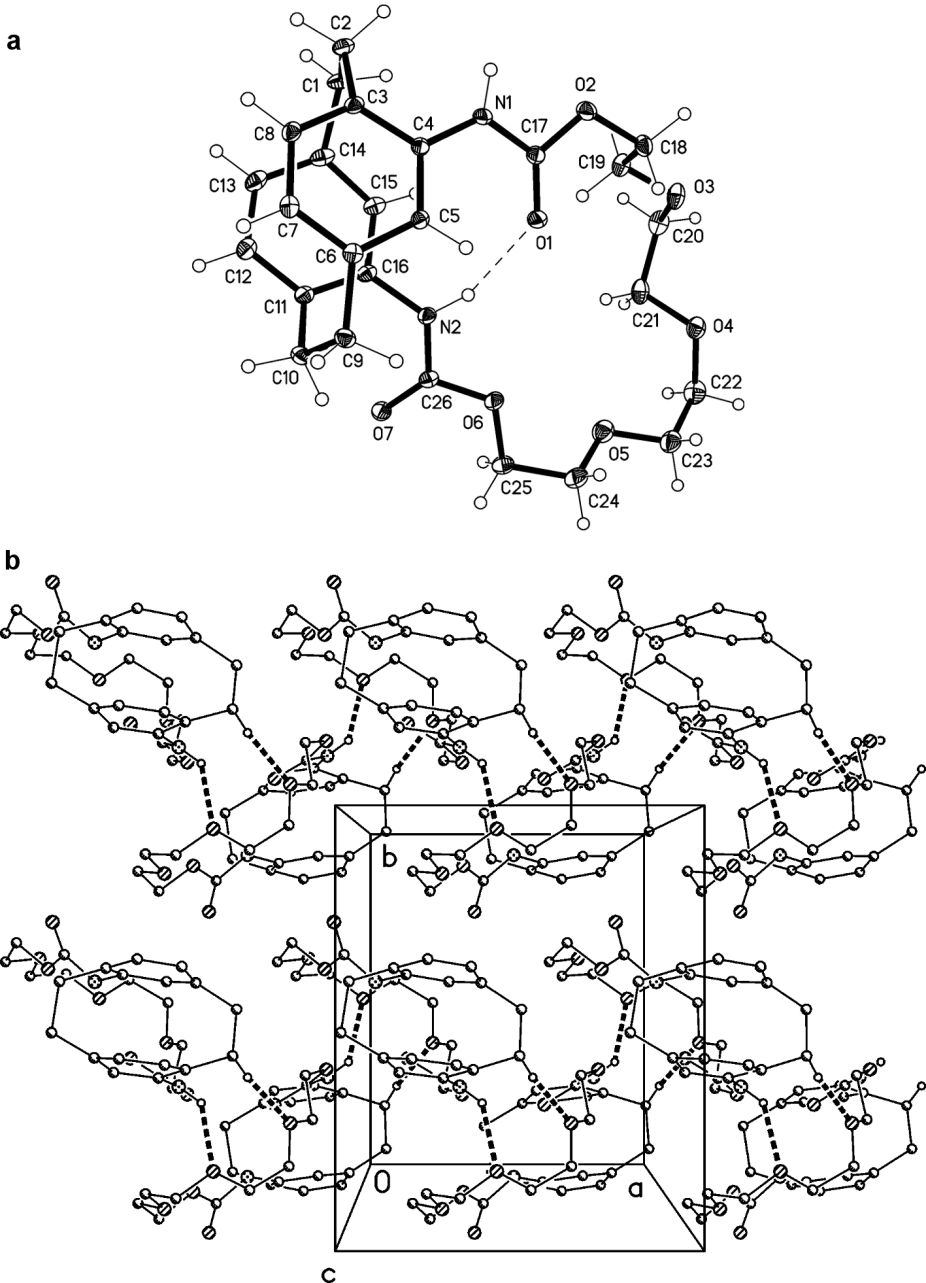
(a, above): The molecule of compound **18** in the crystal; ellipsoids represent 50% probability levels. The intramolecular hydrogen bond is indicated by a dashed line. (b, below): Packing diagram of **18** viewed parallel to the *c* axis in the region *z* ≈ 0. Intermolecular hydrogen bonds (one classical, one "weak") are shown as thick dashed lines. H atoms not involved in these interactions are omitted for clarity.

In the structure of **18** (Figure 3a), the large macrocyclic ring displays an unsymmetrical configuration (e.g., the four O–C–C–O torsion angles starting from O2 are *ap*, ≈ –90°, +*sc*, –*sc*; for detailed values, see the deposited material). This is at least in part connected with the transannular hydrogen bond N2–H02···O1, with H···O 2.07(2) Å and N–H···O 156(2)°. The other NH group is involved in a rather non-linear intermolecular hydrogen bond N1–H01···O4 [H···O 2.28(2) Å, N–H···O 133(2)°], which, together with the "weak" interaction C2–H2A···O3 [H···O 2.47 Å, C–H···O 173°], links the molecules via a 2_1_ screw axis to form columns parallel to the *a*-axis (Figure 3b).

It is a well-known phenomenon, already observed by Cram and Reich in the early days of cyclophane chemistry, that on heating, equilibria are set up between various [2.2]cyclophane isomers [28]. For example, on heating pseudo-*ortho*-disubstituted derivatives, these are converted into their pseudo-*para*-diastereomers.

Carrying out the same experiment with **18**, several days of heating in refluxing triglyme, i.e., at 216 °C, did not, however, furnish the corresponding pseudo-*para*-crownophane, but provided a dark brown intractable material.

Changing the trapping agent for **16** to butane-1,4-diol (toluene, reflux, 20 h) led to bis(urethane diol) **20** (43%). In the hope that this would react with a second equivalent of **16** and yield the cyclic 2:2-adduct **21**, we added more of the bis(isocyanate). Under similar conditions to those above, only polymeric material was obtained, however. We hence assume that intermolecular oligo-/polymerization is favored over the intramolecular process.

**Scheme 3:**
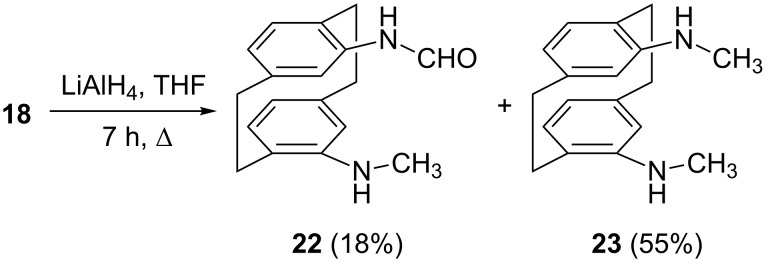
LiAlH_4_-reduction of crownophane **18**.

In order to make the crownophane **18** resemble a “real” crown ether more closely [29], we next tried to reduce its carbonyl groups to methylene units by lithium aluminum hydride (Scheme 3).

Interestingly, the process (reduction in refluxing THF for 7 h) caused a complete destruction of the newly created heterocyclic ring and provided the aldehyde **22** together with the bis secondary amine **23**, the latter in acceptable yields. Although it was not tested specifically, it is likely that **22** is the precursor for **23**.

**Figure 4:**
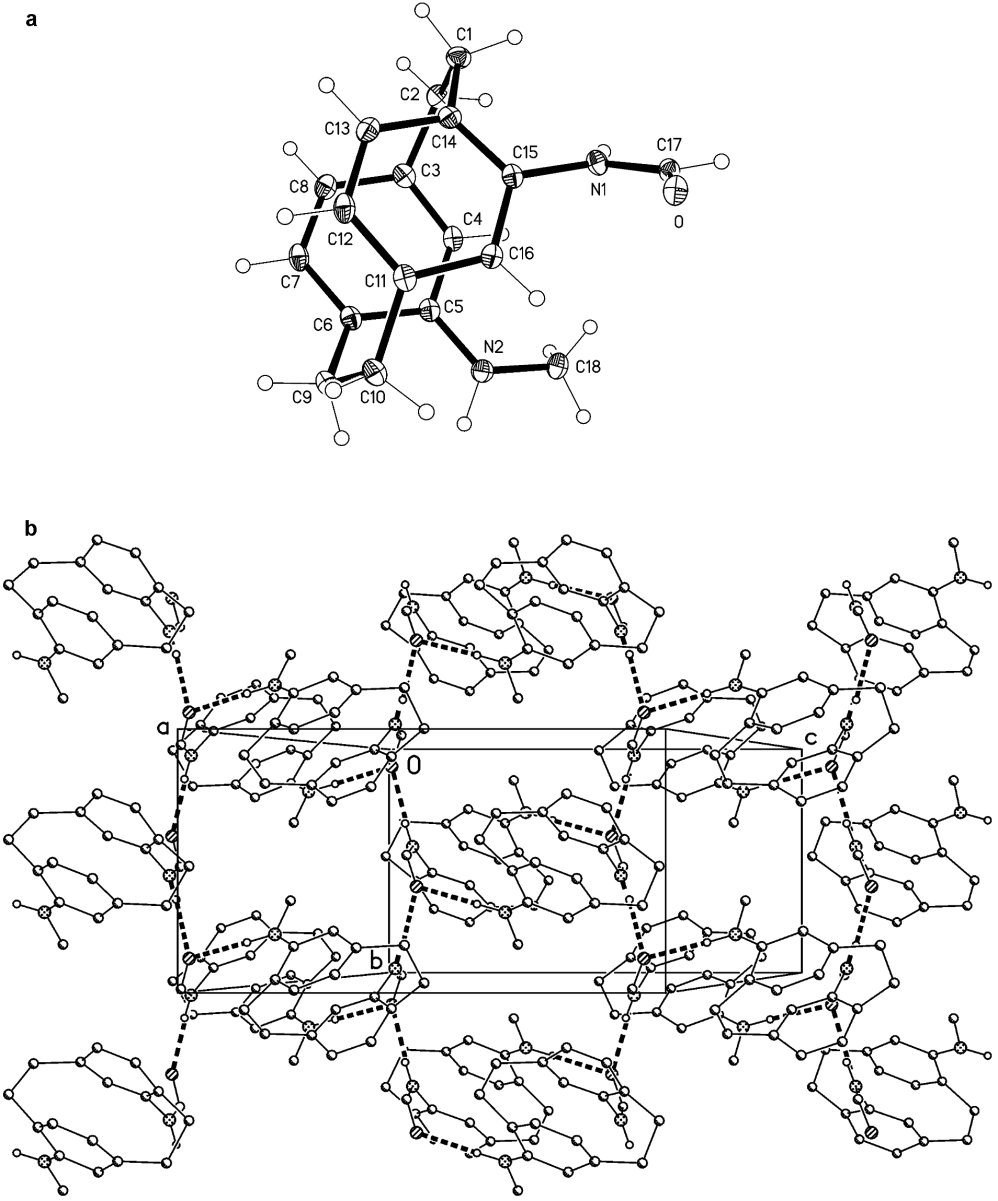
(a, above): The molecule of compound **22** in the crystal; ellipsoids represent 30% probability levels. (b, below): Packing diagram of **22** viewed perpendicular to the *bc*-plane. Classical hydrogen bonds are shown as thick dashed lines. H atoms not involved in these interactions are omitted for clarity.

We assume that the reduction of **18** starts as usual by attack of hydride at the urethane carbonyl group. Because of the inherent strain in the tetrahedral intermediate thus generated, the polyether bridge functions as a leaving group to form an aldehyde intermediate. This is subsequently reduced a second time to the *N*-CH_3_ moiety. In compound **22** one formyl group is still present whereas in **23** this has been lost completely. Both compounds were characterized by their spectroscopic data (Supporting Information File 1), and for **22** we could additionally determine the solid-state structure by X-ray structural analysis (Figure 4a). The orientation of the side chains in **22** is described by the torsion angles C14–C15–N1–C17 131.2(2)°, C15–N1–C17–O −1.3(2)° (the standard *trans*-amide geometry) and C6–C5–N2–C18 176.7(1)°. Both NH groups are involved in classical intermolecular hydrogen bonds. The interaction N2–H02···O [H···O 2.23(2) Å, N–H···O 164(2)°] forms inversion-symmetric dimeric units (easily cognizable in the Figure), which are in turn linked through N1–H01···O [H···O 2.02(2) Å, N–H···O 168(2)°, 2_1_ screw axis parallel to *b*] to form a layer structure parallel to the *bc*-plane (Figure 4b). A very short C–H···π contact involving the formyl hydrogen, C17–H17···Cent (C12,13,15,16)] [normalized H···π 2.50 Å, C–H···π 174°] through the same screw operator supports the hydrogen bonding system but for clarity is not drawn explicitly in Figure 4b.

### Preparation of 4,16-bis(isocyanato)[2.2]paracyclophane (**28**, pseudo-*para-*bis(isocyanate) **28**)

**Scheme 4:**
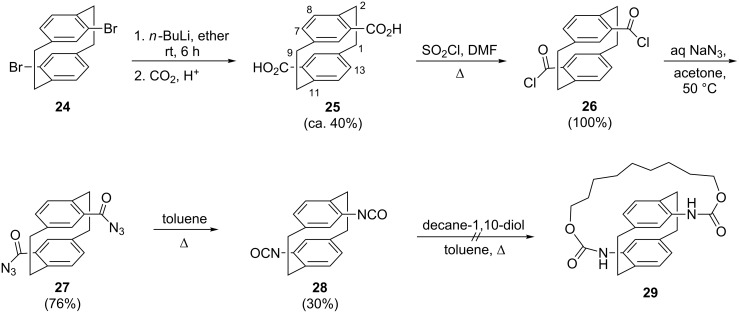
The preparation of several derivatives of 4,16-dicarboxy[2.2]paracyclophane (**25**) carrying *N*-containing functional groups.

With the successful route to **8** and **16** in hand, we next turned our attention to the preparation of several new pseudo-*para*-substituted [2.2]paracyclophane derivatives carrying *N*-functional groups. As shown in Scheme 4, the crucial substrate **25** was prepared from the pseudo-*para*-dibromide **24** [24,29]. Treating **25** with thionyl chloride in DMF yielded the bis(acyl chloride) **26** in quantitative yield. In contrast to most other acyl chlorides, **26** is a stable compound with a high melting point (210 °C), which could easily be recrystallized from dichloromethane/pentane to furnish single crystals suitable for X-structural analysis.

**Figure 5:**
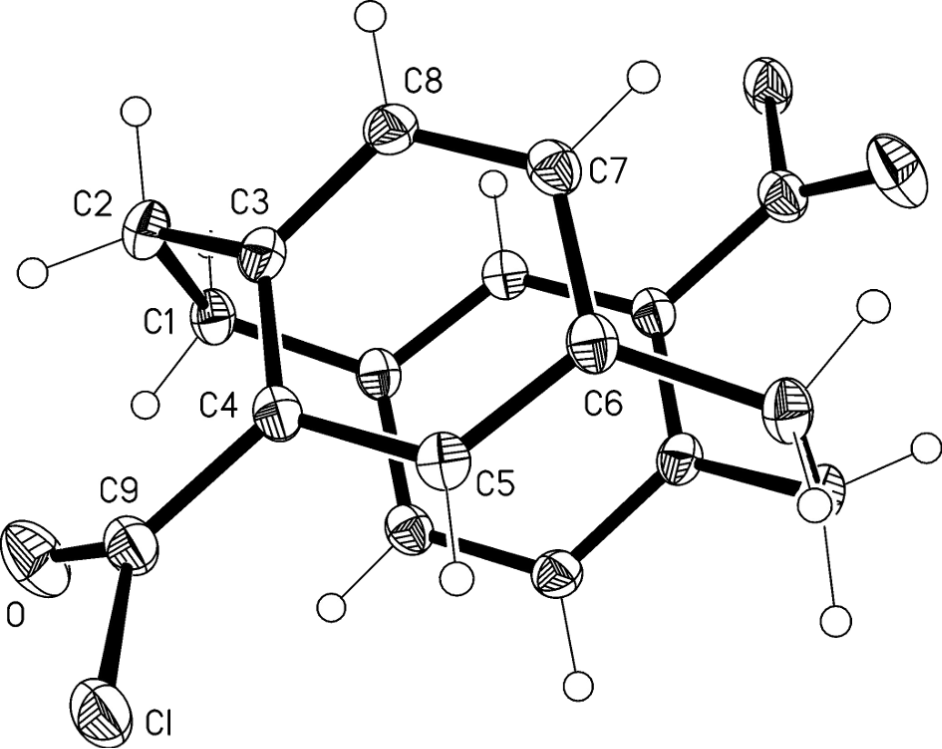
The molecule of compound **26** in the crystal; ellipsoids represent 50% probability levels. Only the asymmetric unit is numbered.

The molecule of **26** (Figure 5) possesses crystallographic inversion symmetry, consistent with its pseudo-*para* form. We have reported the structure of the pseudo-*gem* isomer [20]. The cell constants *b* and *c* of **26**, 11.5213 and 7.5417 Å, respectively, suggest that the packing should display the "7,11"-pattern previously noted by us as being common to a large number of simple cyclophane derivatives; it involves layers of hexagonally arranged molecules, generally with C–H···π contacts [20]. Compound **26** does indeed display the standard pattern, but the sole H···π contact (from H5) is rather long at 2.89 Å (normalized) and indeed there are few other notable intermolecular contacts. The same "7,11"-packing is displayed by compound **28**, for which an explicit packing diagram is presented (see below).

Subjection of **26** to nucleophilic substitution with aqueous sodium azide yielded the bis(keto azide) **27** in 76% yield as a colorless amorphous solid. The compound is readily characterized by its typical azide and carbonyl absorption bands in the vibrational spectral (see Supporting Information File 1 for complete spectroscopic data). The Curtius degradation of **27** provided the desired bis(isocyanate) **28** readily, though in a disappointing isolated yield of 30% only. Since the yield of the crude product was three times as much, we assume that much of the material is lost during chromatography on silica gel. The compound was characterized by its spectroscopic and analytical data and also by the determination of its solid state structure by X-ray diffraction [30].

**Figure 6:**
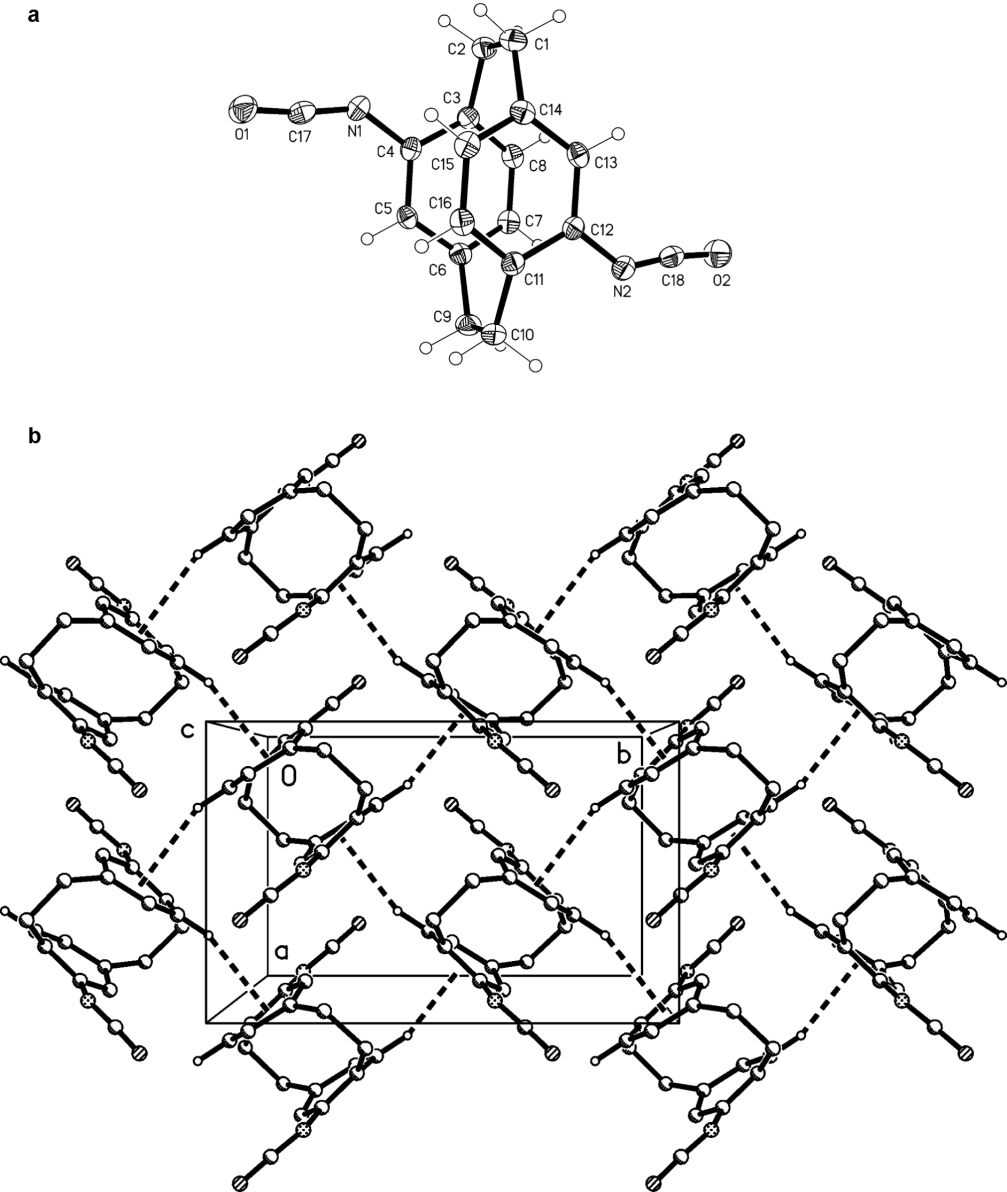
(a, above): The molecule of compound **28** in the crystal; ellipsoids represent 50% probability levels. (b, below): Packing diagram of **28** viewed parallel to the *c*-axis in the region *z* ≈ ¼, showing C–H···π contacts as dashed lines.

The molecule of **28** (Figure 6a) displays no formal crystallographic inversion symmetry, but is approximately inversion-symmetric (rmsd 0.02 Å). Again, we have reported the structure of the corresponding pseudo-*gem* isomer [20]. A search of the Cambridge database [31] revealed, perhaps surprisingly, that there are only two other X-ray structure determinations of organic isocyanates with the NCO group bonded to a phenyl ring. The dimensions of the isocyanate group observed for **28** [C_ring_–N 1.408(2), 1.419(2), N–C 1.194(3), 1.192(3), C–O 1.173(2), 1.174(2) Å; C_ring_–N–O 140.0(2), 136.9(2), N–C–O 172.6(2), 172.5(2)°] are closely similar to the values observed previously. As for **26**, the cell constants *a* 7.2803 and *b* 11.4247 Å suggest that the packing should display the "7,11"-pattern, and this is indeed the case (Figure 6b); the relevant contacts are H16···*Cent*(C4,5,7,8) 2.75 and H8···*Cent*(C12,13,15,16) 2.81 Å. Some borderline C–H···O contacts are also observed (see deposited material).

Of course, compound **28** offers many possibilities for further elaboration. In a first attempt we reacted it with decane-1,10-diol under high dilution conditions in the hope of introducing a bridge between a “lower” and an “upper” deck of a cyclophane, a bridging situation unknown so far. Rather than the intended bis(urethane) **29**, we obtained only intractable material, presumably a mixture of polyurethanes resulting from the α,ω-diol and the bis(isocyanate). It seems reasonable to assume that the future tether reacts once with **28** but then has no time to reorient itself to place the remaining OH function close to the remaining NCO moiety. The subsequent intermolecular pathway is much more likely than the intramolecular cyclization to **29**.

## Conclusion

Having developed different, mostly high-yielding routes to various diamino[2.2]paracyclophanes and related compounds [30], we are now in a position to prepare the tetramines **10** and **11** as the next steps to novel polyfunctionalized cyclophanes.

## Supporting Information

File 1 Experimental and characterization data.
