# Supplementary material for: The preparation of new functionalized [2.2]paracyclophane derivatives with N-containing functional groups
Source: Beilstein J Org Chem. 2015 Apr 7;11:437–45. doi: 10.3762/bjoc.11.50 (PMC4419526; doi:10.3762/bjoc.11.50)
Supplement: File 1 — Experimental and characterization data. [file Beilstein_J_Org_Chem-11-437-s001.pdf]

# Supporting Information

# for

# The preparation of new functionalized [2.2]paracyclophane derivatives with *N*-containing functional groups

**Henning Hopf*[1], Swaminathan Vijay Narayanan[1] and Peter G. Jones[2]**

Address: [1]Institut für Organische Chemie, Technische Universität Braunschweig, Hagenring 30, D-38106 Braunschweig, Germany, Fax: (+49)531-391-5388 and [2]Institut für Anorganische und Analytische Chemie, Technische Universität Braunschweig, Postfach 3329, D-38106 Braunschweig, Germany, Fax: (+49)531-391-5387

Email: Henning Hopf - h.hopf@tu-bs.de

*Corresponding author

Cyclophanes, Part 72 [1]. – For Part 71, see ref. [2]

# Experimental and characterization data

**Instrumentation and general experimental considerations:**

**TLC:** precoated plastic plates, silica gel: Polygram Sil G/$UV_{254}$ (Macherey-Nagel & Co.); alumina: Polygram ALOX N/$UV_{254}$ (Macherey-Nagel & Co.). - **Column Chromatography:** silica gel 60 (70-230 mesh), Merck (Darmstadt). Solvents used for column chromatography were distilled prior to use. - **Melting Points:** Büchi 510 melting point apparatus, MEL-TEMP II (Laboratory Devices, USA), Kofler-Heiztischmikroskop apparatus; melting points are uncorrected. – **NMR:** Bruker AC-200: $^{1}$H NMR (200.1 MHz); $^{13}$C NMR (50.3 MHz); Bruker DRX-400: $^{1}$H NMR (400.1 MHz); $^{13}$C NMR (100.6 MHz). Deuteriochloroform was used as solvent unless otherwise stated. Chemical shifts are reported downfield from the internal tetramethylsilane reference. – **IR:** Nicolet 320 FT-IR spectrometer or Bruker Tensor 27 spectrometer. Samples were prepared as KBr pellets or as thin films. - **UV/Vis:** Varian Cary 100BIO or HP 8452 A Diode Array spectrophotometer. – **MS:** Finnigan MAT 95 spectrometer using electron ionisation (EI, 70 eV). Electron spray ionisation spectra (ESI): Finnigan MAT 95XLT spectrometer. Spray voltage 1.3-1.8 kV, flow rate 0.5-1.5 µL per min. Methanol was used as solvent unless otherwise specified. - **GC/MS:** Finnigan TSQ 700 (EI, 70 eV) attached to Hewlett Packard 5890A gas chromatograph.

**X-Ray structure determinations:**

Numerical data are summarized in Table S1. All compounds

crystallized solvent-free. Crystals were mounted in inert oil on glass fibres and transferred to the cold gas stream of the diffractometer (Bruker SMART 1000 CCD). Intensity data were recorded using monochromated MoKα radiation (λ = 0.71073 Å). No absorption corrections were performed. The structures were refined anisotropically on $F^2$ using the program SHELXL-97 [1]. Hydrogen atoms of NH groups were refined freely (for **18** with N-H distance restraints). Other hydrogens were refined using rigid methyl groups allowed to rotate but not tip, or a riding model starting from calculated positions. Special features: Compounds **18** and **28**, which were racemic or achiral, respectively, crystallize by chance in a chiral (Sohncke) space group. In both cases the anomalous scattering was insignificant, and the Friedel opposite reflections were therefore averaged, making the Flack parameter meaningless. For **26**, there is no evidence for disorder by mixing of O and Cl sites.

Crystallographic data have been deposited with the Cambridge Crystallographic Data Centre as supplementary publications no. CCDC-1033658 (**18**), - 1033659 (**22**), - 1033660 (**26**), - 1033661 (**28**). Copies of the data can be obtained free of charge from www.ccdc.cam.ac.uk/data_request/cif.

Nitrogen and argon gas were used for maintaining inert atmosphere. All solvents and chemicals were purified as prescribed in Purification of Laboratory Chemicals, 4th edition, Butterworth-Heinemann, Oxford 2000. – The diacids **14**

[2,3] and **25** [4,5] were prepared as described in the literature.

**4,12-Bis(chlorocarbonyl)[2.2]paracyclophane and 4,12-bis(azido-carbonyl)[2.2]paracyclophane (15):**

(a) To a suspension of 4,12-dicarboxy[2.2]paracyclophane (**14**, 0.80 g, 2.70 mmol) in anhydrous $CH_2Cl_2$ (8 mL) was added oxalyl-chloride (8.0 mL) and a drop of a 10% mixture of $CH_2Cl_2$/DMF (v/v) at room temp. The reaction mixture was stirred at room temp. for ca. 3 h until it became clear. Then benzene (10 mL) was added, and the solvents were evaporated under reduced pressure. The crude product (0.90 g, 100% yield) was used for further synthesis.

(b) To a suspension of the 4,12-dicarboxy[2.2]paracyclophane (**14**, 0.16 g, 0.54 mmol) in $SOCl_2$ (7 mL) were added under nitrogen 2 drops of DMF, and the mixture was refluxed for 45 min. The reaction mixture became clear. The solvent was removed under reduced pressure and the crude pale yellow solid (0.18 g, 0.53 mmol, 98%) was dried under high vacuum. The crude product was used for further synthesis but resulted in lower yields than from method (a). - m. p.: 95 °C. - **$^{1}$H NMR** (200.1 MHz, $CDCl_3$): δ = 6.95 (d, 2 H, $J_{5\text{-H},7\text{-H}} = J_{15\text{-H},13\text{-H}} = 1.4$ Hz, 5-H, 15-H), 6.66 (dd, 2 H, $J_{7\text{-H},5\text{-H}} = J_{13\text{-H},15\text{-H}} = 1.5$ Hz, $J_{7\text{-H},8\text{-H}} = J_{13\text{-H},12\text{-H}} = 6.2$ Hz, 7-H, 13-H), 6.63 (d, 2 H, $J_{8\text{-H},7\text{-H}} = J_{12\text{-H},13\text{-H}} = 6.6$ Hz, 8-H, 12-H), 3.72-3.46 (m, 2 H, bridge-H), 3.30-2.97 ppm (m, 4 H, bridge-H), 2.58-2.51 ppm (m, 2 H, bridge-H). - **$^{13}$C**

**NMR** (50.3 MHz, $CDCl_3$): δ = 139.6, 136.8, 135.9, 134.7, 129.1, 34.2, 33.3 ppm; one aromatic carbon atom could not be detected and the carbonyl carbon atom was too weak to be registered.

**4,12-Bis(azidocarbonyl)[2.2]paracyclophane (15):** To a suspension of the crude 4,12-bis(chlorocarbonyl)[2.2]paracyclophane (0.18 g, 0.54 mmol) in acetone (15 mL) was added dropwise at 0 °C an aqueous solution of $NaN_3$ (0.35 g, 5.38 mmol) in water (10 mL), and the mixture was stirred at the same temp. for 1 h. Then ice cold water (50 mL) was added and the precipitate was filtered off; the residue was washed several times with ice cold water and allowed to air dry. The crude pale yellow amorphous solid (0.17 g) was obtained in 90% yield. - **m. p.**: no sharp melting point, as compound gradually decomposes on heating. - **$^1$H NMR** (200.1 MHz, $CDCl_3$): δ = 7.16 (d, 2 H, $J_{5\text{-H},7\text{-H}}$ = $J_{15\text{-H},13\text{-H}}$ = 1.8 Hz, 5-H, 15-H), 6.78 (dd, 2 H, $J_{7\text{-H},5\text{-H}}$ = $J_{13\text{-H},15\text{-H}}$ = 1.8 Hz, $J_{7\text{-H},8\text{-H}}$ = $J_{13\text{-H},12\text{-H}}$ = 7.9 Hz, 7-H, 13-H), 6.59 (d, 2 H, $J_{8\text{-H},7\text{-H}}$ = $J_{12\text{-H},13\text{-H}}$ = 7.9 Hz, 8-H, 12-H), 4.24–4.12 (m, 2 H, bridge-H), 3.29–3.07 (m, 4 H, bridge-H), 2.93–2.78 ppm (m, 2 H, bridge-H). - **$^{13}$C NMR** (50.3 MHz, $CDCl_3$): δ = 143.4 (s, Ar), 140.6 (s, Ar), 137.7 (d, Ar), 136.4 (d, Ar), 133.5 (d, Ar), 130.0 (s, Ar), 35.7 (t, bridge), 33.9 ppm (t, bridge); due to weak intensity the carbonyl peak was not detected. - **IR** (KBr): nu(tilde) = 3050 (w), 2968 (w), 2930 (w), 2855 (w), 2268 (m), 2141 (s), 1680 (s), 1656 (w), 1247 (vs), 1199 (s), 1189 (s), 1155 (m), 994 (s), 917 $cm^{-1}$ (m). - **MS** (EI, 70 eV): *m/z* (%) =

346 (2) [$M^+$], 290 (20), 145 (100), 116 (9), 90 (15), 51 (5), 44 (9). - **HRMS**: not available due to very low intensity of [$M^+$].

**4,12-Diisocyanato[2.2]paracyclophane (16)**: The crude 4,12-bis-(azidocarbonyl)[2.2]paracyclophane (**15**, 1.44 g, 4.16 mmol) was taken up in dry toluene (50 mL) under $N_2$ and the mixture refluxed for 30 min. The solvent was evaporated *in vacuo* and the crude residue was column chromatographed on silica gel with $CH_2Cl_2$/pentane (1:4, v/v) to yield the pseudo-*ortho*-diisocyanato-[2.2]paracyclophane (**16**, 1.20 g, 100%) as a colorless solid. - **m. p.**: 94-96 °C. - **$^1$H NMR** (400.1 MHz, $CDCl_3$): δ = 6.51 (d, 2 H, $J_{8-H,7-H}$ = $J_{12-H,13-H}$ = 7.91 Hz, 8-H, 12-H), 6.49 (d, 2 H, $J_{5-H,7-H}$ = $J_{15-H,13-H}$ = 1.75 Hz, 5-H, 15-H), 6.40 (dd, 2 H, $J_{7-H,5-H}$ = $J_{13-H,15-H}$ = 1.75 Hz, $J_{7-H,8-H}$ = $J_{13-H,12-H}$ = 7.91 Hz, 7-H, 13-H), 3.34–3.27 (m, 2 H, 2-H, 10-H), 3.09–2.95 (m, 4 H, 1-H, 9-H), 2.77–2.69 ppm (m, 2 H, 2-H, 10-H). - **$^{13}$C NMR** (100.6 MHz, $CDCl_3$): δ = 181.3 (s, C-18, C-21), 141.6 (s, C-6, C-14), 135.1 (d, C-8, C-12), 133.8 (s, C-3, C-11), 133.4 (s, C-4, C-16), 130.3 (d, C-7, C-13), 126.6 (d, C-5, C-15), 33.3 (t, C-1, C-9), 32.2 ppm (t, C-2, C-10). - **IR** (KBr): nu(tilde) = 3047 (w), 2855 (w), 2296 (NCO, vs), 1560 (w), 1505 (w), 872 $cm^{-1}$ (w). - **UV/Vis** ($CHCl_3$): $\lambda_{max}$ (lg ε) = 310 (2.67), 292 nm (2.95). - **MS** (EI, 70 eV): *m/z* (%) = 290 (24) [$M^+$], 276 (2), 249 (10), 145 (100), 138 (20), 116 (9), 104 (28), 90 (12). - **HRMS** ($C_{18}H_{14}N_2O_2$) calcd.: 290.105527, found: 290.10542 ± 1 ppm.

**4,12-Diamino[2.2]paracyclophane (8):** 4,12-Diisocyanato[2.2]-paracyclophane (**16**, 0.10 g, 0.345 mmol) was taken up in ethanol (10 mL) and the mixture was refluxed for 2 h. To this solution was added 20% aq. KOH solution (5 mL) and the mixture was refluxed for 45 h with stirring. To the cooled reaction mixture was added 20% aq. KOH solution (30 mL), and the mixture was stirred for 5 min. The precipitate was removed by filtration, washed several times with water, and dried ($Na_2SO_4$), yielding the pseudo-*ortho*-diamino[2.2]paracyclophane (**8**, 0.08 g, 98%) as a pale brown solid. - **m. p.**: >200 °C (decomp.). - $^{1}$**H NMR** (400.1 MHz, $CDCl_3$): δ = 6.28 (d, 2 H, $J_{8\text{-H},7\text{-H}}$ = $J_{12\text{-H},13\text{-H}}$ = 7.69 Hz, 8-H, 12-H), 6.11 (d, 2 H, $J_{5\text{-H},7\text{-H}}$ = $J_{15\text{-H},13\text{-H}}$ = 1.57 Hz, 5-H, 15-H), 5.96 (dd, 2 H, $J_{7\text{-H},8\text{-H}}$ = $J_{13\text{-H},12\text{-H}}$ = 7.69 Hz, $J_{7\text{-H},5\text{-H}}$ = $J_{13\text{-H},15\text{-H}}$ = 1.63 Hz, 7-H, 13-H), 3.32 (br. s, 4 H, 17-H, 18-H), 2.99–2.93 (m, 2 H, 2-H, 10-H), 2.88–2.78 (m, 4 H, 1-H, 9-H), 2.58–2.50 ppm (m, 2 H, 2-H, 10-H). - $^{13}$**C NMR** (100.6 MHz, $CDCl_3$): δ = 144.5 (s, C-4, C-16), 141.1 (s, C-6, C-14), 135.1 (d, C-8, C-12), 124.1 (s, C-3, C-11), 123.1 (d, C-7, C-13), 116.3 (d, C-5, C-15), 32.7 (t, C-1, C-9), 32.0 ppm (t, C-2, C-10). - **IR** (diamond-ATR): nu(tilde) = 3368 (w), 2923 (w), 1608 (m), 981 (w), 784 $cm^{-1}$ (w). - **UV/Vis** ($CH_3CN$): $\lambda_{max}$ (lg ε) = 321 (3.08), 274 nm (3.50). - **MS** (EI, 70 eV): *m/z* (%) = 238 (36) [$M^+$], 119 (100), 91 (12), 44 (4). - **HRMS** ($C_{16}H_{18}N_2$) calcd.: 238.14699, found: 238.14702 ± 1.5 ppm.

**Pseudo-*ortho*-crownophane 18**: Pseudo-*ortho*-diisocyanato-[2.2]-paracyclophane **16** (0.07 g, 0.24 mmol) was taken up in anhydrous toluene (100 mL) under $N_2$ with stirring. The reaction mixture was heated to reflux and a solution of tetra-ethyleneglycol (0.046 g, 0.24 mmol) in toluene (50 mL) was added slowly (ca. 1.2 mL/h). The reaction mixture was refluxed with stirring for 7 d, the solvent was evaporated under vacuum, and the crude product was subjected to column chromatography on silica gel with hexane/EtOAc (3:7), yielding (0.08 g, 68%) of crownophane **18**) as a colorless solid. Plate-like single crystals for X-ray analysis were obtained by slow diffusion of pentane into the solution of **18** in $CH_2Cl_2$. - **m. p.**: 176 °C. - **$^{1}$H NMR** (400.1 MHz, $CDCl_3$): δ = 7.3 (br. s, 2 H, NH, 17-H, 35-H), 6.73 (d, 2 H, $J_{5\text{-H},7\text{-H}}$ = $J_{15\text{-H},13\text{-H}}$ = 1.23 Hz, 5-H, 15-H), 6.51 (d, 2 H, $J_{8\text{-H},7\text{-H}}$ = $J_{12\text{-H},13\text{-H}}$ = 7.84 Hz, 8-H, 12-H), 6.38 (dd, 2 H, $J_{7\text{-H},5\text{-H}}$ = $J_{13\text{-H},15\text{-H}}$ = 1.64 Hz, $J_{7\text{-H},8\text{-H}}$ = $J_{13\text{-H},12\text{-H}}$ = 7.83 Hz, 7-H, 13-H), 4.63 (br., 2 H, 21-H, 31-H), 4.16 (br. s, 2 H, 21-H, 31-H), 3.82–3.62 (m, 12 H, 22-H, 24-H, 25-H, 27-H, 28-H, 30-H,), 3.30–3.24 (m, 2 H, 2-H, 10-H), 3.06–2.91 (m, 4 H, 1-H, 9-H), 2.79–2.72 ppm (m, 2 H, 2-H, 10-H). - **$^{13}$C NMR** (100.6 MHz, $CDCl_3$): δ = 154.3 (s, C-18, C-33), 140.7 (s, C-4, C-16), 136.1 (s, C-6, C-14), 135.3 (d, C-8, C-12), 132.1 (s, C-3, C-11), 129.6 (d, C-7, C-13), 123.1 (d, C-5, C-15), 70.7, 70.5, 69.5, 63.9 (t, C-21, C-22, C-24, C-25, C-27, C-28, C-30, C-31), 33.3 (t, C-1, C-9), 32.6 ppm (t, C-2, C-10). - **IR**

(KBr): nu(tilde) = 3256 (w), 2893 (w), 1738 (vs), 1719 (s), 1687 (vs), 1548 (m), 1531 (m), 1245 (s), 1231 (s), 1131 (w), 1103 (m), 1087 $cm^{-1}$ (w). - **UV/Vis** ($CHCl_3$): $\lambda_{max}$ (lg ε) = 314 (2.54), 300 (2.75), 290 (3.03), 268 nm (3.45). - **MS** (EI, 70 eV): *m/z* (%) = 484 (32) [$M^+$], 396 (6), 290 (28), 190 (20), 167 (11), 149 (20), 145 (100), 119 (10), 89 (13), 71 (10), 57 (15), 45 (19). - **HRMS** ($C_{26}H_{32}N_2O_7$) calcd.: 484.220951, found: 484.22096 ± 1 ppm.

**Pseudo-*ortho*-crownophane 19:** Pseudo-*ortho*-diisocyanato[2.2]-paracyclophane (**16**, 0.07 g, 0.24 mmol) was taken up in anhydrous toluene (100 mL) under $N_2$ with stirring. The reaction mixture was heated to reflux and a solution of pentaethyleneglycol (0.057 g, 0.24 mmol) toluene (50 mL) was added slowly (1.2 mL/h). After refluxing with stirring for 7 d, the solvent was evaporated *in vacuo* and the crude product was subjected to column chroma-tography on neutral alumina with hexane/EtOAc (1:1), yielding 0.09 g (73%) of crownophane **19** as a colorless solid. - **m. p.**: 147 °C. - **$^1$H NMR** (400.1 MHz, $CDCl_3$): δ = 7.35 (br. s, 2 H, NH, 17-H, 38-H), 6.70 (2 H, 5-H, 15-H), 6.51 (d, 2 H, $J_{8-H,7-H}$ = $J_{12-H,13-H}$ = 7.84 Hz, 8-H, 12-H), 6.38 (dd, 2 H, $J_{7-H,5-H}$ = $J_{13-H,15-H}$ = 1.43 Hz, $J_{7-H,8-H}$ = $J_{13-H,12-H}$ = 7.83 Hz, 7-H, 13-H), 4.53 (2 H, 21-H, 34-H), 4.26 (2 H, 21-H, 34-H), 3.84–3.64 (m, 16 H, 22-H, 24-H, 25-H, 27-H, 28-H, 30-H, 31-H, 33-H), 3.33–3.27 (m, 2 H, 2-H, 10-H), 3.07–2.90 (m, 4 H, 1-H, 9-H), 2.78–2.08 ppm (m, 2 H, 2-H, 10-H). - **$^{13}$C NMR** (100.6

MHz, $CDCl_3$): δ = 154.2 (s, C-18, C-36), 140.7 (s, C-4, C-16), 136.02 (s, C-6, C-14), 135.5 (d, C-8, C-12), 132.2 (s, C-3, C-11), 129.7 (d, C-7, C-13), 123.2 (d, C-5, C-15), 70.89, 70.82, 70.76, 69.5 (t, C-22, C-24, C-25, C-27, C-28, C-30, C-31, C-33), 64.4 (t, C-21, C-34), 33.2 (t, C-1, C-9), 32.9 ppm (t, C-2, C-10). - **IR** (KBr): nu(tilde) = 3436 (w), 3330 (s), 3312 (s), 2947 (s), 2927 (s), 2881 (s), 2866 (s), 1727 (vs), 1704 (vs), 1599 (s), 1574 (s), 1533 (vs), 1519 (vs), 1493 (vs), 1454 (s), 1435 (s), 1422 (m), 1294 (s), 1245 (vs), 1229 (vs), 1223 (vs), 1199 (m), 1130 (s), 1102 (vs), 1073 (vs), 1044 (m), 1032 (m), 879 $cm^{-1}$ (m). - **UV/Vis** ($CH_3CN$): $\lambda_{max}$ (lg ε) = 268 (3.72), 236 nm (4.12). - **MS** (EI, 70 eV): *m/z* (%) = 528 (41) [$M^+$], 440 (6), 396 (5), 352 (6), 308 (5), 290 (26), 190 (41), 145 (100), 119 (16), 89 (26), 55 (11). - **HRMS** ($C_{28}H_{36}N_2O_8$) calcd.: 528.247166, found: 528.24515 ± 1 ppm.

**4,12-Bis-urethane diol 20**: A dried, $N_2$-flushed three-necked flask with reflux condenser and a dropping funnel was charged with 1,4-butanediol (0.12 g, 1.36 mmol) along with anhydrous toluene (50 mL). The stirred reaction mixture was heated to reflux, and 4,12-diisocyanato[2.2]paracyclophane (**16**, 0.10 g, 0.35 mmol) in toluene (15 mL) was added slowly. The reaction mixture was refluxed for 20 h, cooled, and the solvent was evaporated *in vacuo*. The crude product was column chromatographed on silica gel with ethyl acetate yielding the desired **20** (0.07 g, 43%) as a colorless solid. - **m. p.**: 60 °C. - **$^1$H NMR** (400.1 MHz, $CDCl_3$): δ = 7.31 (br. s, 2 H, NH, 17-H,

26-H), 6.69 (2 H, 5-H, 15-H), 6.50 (d, 2 H, $J_{8\text{-H},7\text{-H}}$ = $J_{13\text{-H},12\text{-H}}$ = 7.84 Hz, 8-H, 13-H), 6.38 (dd, 2 H, $J_{7\text{-H},5\text{-H}}$ = $J_{13\text{-H},15\text{-H}}$ = 1.35 Hz, $J_{7\text{-H},8\text{-H}}$ = $J_{13\text{-H},12\text{-H}}$ = 7.83 Hz, 7-H, 13-H), 4.29–4.20 (m, 4 H, 21-H, 30-H), 3.71–3.65 (m, 4 H, 24-H, 33-H), 3.32–3.26 (m, 2 H, 2-H, 10-H), 3.08–3.03 (m, 2 H, 1-H, 9-H), 2.96–2.88 (m, 2 H, 1-H, 9-H), 2.77–2.69 (m, 2 H, 2-H, 10-H), 2.17 (br. s, 2 H, OH, 25-H, 34-H), 1.82–1.65 ppm (m, 8 H, 22-H, 23-H, 31-H, 32-H). – **$^{13}$C NMR** (100.6 MHz, $CDCl_3$): δ = 154.6 (s, C-18, C-27), 140.6 (s, Ar), 136.1 (s, Ar), 135.5 (d, C-8, C-12), 129.5 (d, C-7, C-13), 123.0 (d, C-5, C-15), 65.2 (t, C-21, C-30), 62.2 (t, C-24, C-33), 33.2 (t, C-1, C-9), 32.9 (t, C-2, C-10), 29.1 (t, C-23, C-32), 25.5 (t, C-22, C-31); one carbon atom not resolved due to extended line broadening. – **IR** (KBr): nu(tilde) = 3313 (s), 3301 (s), 2944 (s), 2869 (m), 1704 (vs), 1599 (s), 1572 (s), 1529 (vs), 1494 (vs), 1457 (s), 1439 (m), 1422 (s), 1407 (m), 1292 (s), 1232 (vs), 1199 (w), 1060 (s), 953 (w), 880 (w) $cm^{-1}$. – **UV/Vis** ($CH_3CN$): $\lambda_{max}$ (lg ε) = 268 (3.68), 234 nm (4.14). – **MS** (EI, 70 eV): *m/z* (%) = 470 (12) [$M^+$], 380 (12), 354 (19), 308 (24), 290 (19), 264 (12), 238 (10), 163 (29), 146 (86), 145 (100), 119 (48), 91 (13), 55 (13), 42 (14). – **HRMS** ($C_{26}H_{34}N_2O_6$) calcd.: 470.241687, found: 470.24153 ± 2 ppm.

**Reduction of 18 with lithium aluminium hydride**: A dried, $N_2$-flushed, two-necked flask (100 mL), with reflux condenser and stirrer, was charged with crownophane **18** (0.10 g, 0.21 mmol)

along with anhydrous THF (50 mL). To this solution $LiAlH_4$ (0.039 g, 1.03 mmol) was added with stirring (note: highly exothermic process). The reaction mixture was refluxed for 7 h, cooled to room temp., and finally quenched by adding water. The THF was removed in a rotary evaporator, and the water phase was extracted with $CH_2Cl_2$. The organic phase was washed with aq. sat. $NaHCO_3$, brine, dried ($MgSO_4$) and the solvent was evaporated *in vacuo*. The crude product was subjected to flash column chromatography on silica gel with EtOAc/hexane (7:3), yielding **23** (0.030 g, 55%) and **22** (0.011 g, 18%), both as colorless solids. Compound **22** was recrystallized from $CH_2Cl_2$/pentane to give tablet-shaped single crystals. During the whole reaction and workup procedure exposure to light was avoided. The compounds were characterized immediately, as on exposure to light they decomposed steadily.

**Pseudo-*ortho*-(*N*-methyl)(*N'*-methyl)diamino[2.2]paracyclophane (23): m. p.**: 175-177 °C. - $^{1}$**H NMR** (400.1 MHz, $CDCl_3$): δ = 6.26 (d, 2 H, $J_{8\text{-H},7\text{-H}}$ = $J_{12\text{-H},13\text{-H}}$ = 7.49 Hz, 8-H, 12-H), 5.91 (dd, 2 H, $J_{7\text{-H},8\text{-H}}$ = $J_{13\text{-H},12\text{-H}}$ = 7.54 Hz, $J_{7\text{-H},5\text{-H}}$ = $J_{13\text{-H},15\text{-H}}$ = 1.66 Hz, 7-H, 13-H), 5.88 (d, 2 H, $J_{5\text{-H},7\text{-H}}$ = $J_{15\text{-H},13\text{-H}}$ = 1.51 Hz, 5-H, 15-H), 3.43 (br. s, 2 H, 17-H, 19-H), 2.98–2.82 (m, 6 H, 1-H, 2-H, 9-H, 10-H), 2.66 (s, 6 H, 18-H, 20-H), 2.59–2.52 ppm (m, 2 H, 2-H, 10-H). - $^{13}$**C NMR** (100.6 MHz, $CDCl_3$): δ = 147.7 (s, C-4, C-16), 141.4 (s, C-6, C-14), 134.6 (d, C-8, C-12), 123.3 (s, C-3, C-11), 121.1 (d, C-7, C-13), 111.7 (d, C-5, C-15), 33.1 (t, C-1,

C-9), 32.4 (t, C-2, C-10), 30.5 ppm (q, C-18, C-20). - **IR** (diamond-ATR): nu(tilde) = 3370 (w), 2922 (m), 2854 (m), 1593 (s), 1567 (s), 1505 (s), 1443 (s), 1407 (s), 1055 (w), 846 $cm^{-1}$ (w). - **UV/Vis** ($CH_3CN$): $\lambda_{max}$ (lg ε) = 306 (3.44), 278 nm (3.75). - **MS** (EI, 70 eV): *m/z* (%) = 266 (25) [$M^+$], 133 (100), 125 (12), 111 (24), 97 (31), 83 (30), 69 (40), 57 (52), 44 (8). - **HRMS** ($C_{18}H_{22}N_2$) calcd.: 266.17829, found: 266.17835 ± 1 ppm.

**Pseudo-*ortho*-(*N*-formyl)(*N'*-methyl)diamino[2.2]paracyclophane (22): m. p.**: 194 °C. - **$^1$H NMR** (400.1 MHz, $CDCl_3$): δ = 8.37 (d, 1 H, $J_{18\text{-H},17\text{-H}}$ = 11.47 Hz, 18-H), 7.24 (br. s, 1 H, 17-H), 6.58 (d, 1 H, $J_{5\text{-H},7\text{-H}}$ = 1.40 Hz, 5-H), 6.51 (d, 1 H, $J_{8\text{-H},7\text{-H}}$ = 7.83 Hz, 8-H), 6.31 (dd, 1 H, $J_{7\text{-H},8\text{-H}}$ = 7.83 Hz, $J_{7\text{-H},5\text{-H}}$ = 1.57 Hz, 7-H), 6.27 (d, 1 H, $J_{12\text{-H},13\text{-H}}$ = 7.65 Hz, 12-H), 5.99 (dd, 1 H, $J_{13\text{-H},12\text{-H}}$ = 7.63 Hz, $J_{13\text{-H},15\text{-H}}$ = 1.61 Hz, 13-H), 5.68 (d, 1 H, $J_{15\text{-H},13\text{-H}}$ = 1.54 Hz, 15-H), 3.40 (br. s, 1 H, 19-H), 3.17–2.51 (m, 8 H, 1-H, 2-H, 9-H, 10-H), 2.68 ppm (s, 3 H, 20-H). - **$^{13}$C NMR** (100.6 MHz, $CDCl_3$): δ = 163.2 (d, C-18), 147.9 (s, C-14/16), 141.5 (s, C-4/6), 141.3 (s, C-14/16), 135.5 (d, C-8), 135.2 (d, C-12), 134.8 (s, C-4/6), 130.1 (d, C-7), 129.1 (s, C-3), 123.9 (s, C-11), 121.3 (d, C-13), 119.5 (d, C-5), 112.1 (d, C-15), 33.7 (t, C-1), 33.1 (t, C-9), 32.1 (t, C-10), 31.9 (t, C-2), 30.9 ppm (q, C-20). - **IR** (diamond-ATR): nu(tilde) = 3412 (w), 2852 (w), 1662 (m), 1634 (m), 1565 (m), 1514 (s), 1249 (w), 889 (w), 714 $cm^{-1}$ (m). - **UV/Vis** ($CH_3CN$): $\lambda_{max}$ (lg ε) = 278 nm (3.72). - **MS** (EI, 70 eV): *m/z* (%) = 280 (24) [$M^+$], 133 (100), 104 (15),

91 (12), 78 (12), 44 (5). - **HRMS** ($C_{18}H_{20}N_2O$) calcd.: = 280.15756, found: 280.15641 ± 1 ppm.

**4,16-Bis(chlorocarbonyl)[2.2]paracyclophane (26):** To a suspension of 4,16-dicarboxy[2.2]paracyclophane (**25**, 0.40 g, 1.35 mmol) in $SOCl_2$ (5 mL) under $N_2$ was added a drop of DMF, and the mixture was refluxed until the solution became clear. The solvent was removed under high vacuum to yield (0.45 g, 100%) pseudo-*para*-bis(chlorocarbonyl)[2.2]paracyclophane (**26**) as a pale yellow solid. Recrystallization from $CH_2Cl_2$/pentane by the slow diffusion method yielded tablet-shaped single crystals used for X-ray analysis. - **m. p.**: 210 °C. - **$^1$H NMR** (400.1 MHz, $CDCl_3$): δ = 7.40 (d, 2 H, $J_{5-H,7-H}$ = $J_{13-H,15-H}$ = 1.83 Hz, 5-H, 13-H), 6.87 (dd, 2 H, $J_{7-H,5-H}$ = $J_{15-H,13-H}$ = 1.91 Hz, $J_{7-H,8-H}$ = $J_{15-H,16-H}$ = 7.82 Hz, 7-H, 15-H), 6.58 (d, 2 H, $J_{8-H,7-H}$ = $J_{16-H,15-H}$ = 7.84 Hz, 8-H, 16-H), 3.93–3.86 (m, 2 H, 2-H, 10-H), 3.27–3.23 (m, 4 H, 1-H, 9-H), 3.03–2.96 ppm (m, 2 H, 2-H, 10-H). - **$^{13}$C NMR** (100.6 MHz, $CDCl_3$): δ = 167.4 (s, C-17, C-18), 143.7 (s, C-3, C-11), 140.9 (s, C-4, C-12), 137.9 (d, C-5, C-13), 136.7 (d, C-7, C-15), 135.4 (d, C-8, C-16), 134.0 (s, C-6, C-14), 34.9 (t, C-2, C-10), 33.8 ppm (t, C-1, C-9). - **IR** (KBr): nu(tilde) = 3017 (w), 2940 (w), 1765 (s), 1677 (vs), 1324 (w), 1305 (m), 1278 (m), 1194 (w), 799 $cm^{-1}$ (w). - **UV/Vis** ($CH_3CN$): $\lambda_{max}$ (lg ε) = 316 (2.86), 264 nm (3.59). - **MS** (EI, 70 eV): *m/z* (%) = 332 (25) [$M^+$], 298 (20), 296 (45), 268 (15), 247 (14), 166 (29),

138 (14), 131 (100), 103 (44), 77 (39), 51 (12). - **HRMS** ($C_{18}H_{14}O_2Cl_2$) calcd.: 332.03708, found: 332.03616 ± 1 ppm.

**4,16-Bis(azidocarbonyl)[2.2]paracyclophane (27):** To a suspension of 4,16-bis(chlorocarbonyl)[2.2]paracyclophane (**26**, 0.42 g, 1.265 mmol) in acetone (15 mL) was slowly added a solution of sodium azide (0.82 g, 12.65 mmol) in water (15 mL) over 10 min. The reaction mixture was warmed to 50 °C and stirring continued at this temperature for 5 h. For work-up, ice cold water (100 mL) was added, then the precipitate was filtered off and air-dried to give **27** (0.33 g, 76%) as colorless amorphous material. - **m. p.**: 110 °C (decomp.). - **$^1$H NMR** (200.1 MHz, $CDCl_3$): δ = 7.15 (d, 2 H, $J_{5-H,7-H}$ = $J_{13-H,15-H}$ = 1.9 Hz, 5-H, 13-H), 6.75 (dd, 2 H, $J_{7-H,5-H}$ = $J_{15-H,13-H}$ = 1.9 Hz, $J_{7-H,8-H}$ = $J_{15-H,16-H}$ = 7.8 Hz, 7-H, 15-H), 6.52 (d, 2 H, $J_{8-H,7-H}$ = $J_{16-H,15-H}$ = 7.8 Hz, 8-H, 16-H), 4.20–4.08 (m, 2 H, bridge-H), 3.23–3.00 (m, 4 H, bridge-H), 2.96–2.85 ppm (m, 2 H, bridge-H). - **$^{13}$C NMR** (50.3 MHz, $CDCl_3$): δ = 143.3 (s, Ar), 141.0 (s, Ar), 136.0 (d, Ar), 135.6 (d, Ar), 135.1 (d, Ar), 131.0 (s, Ar), 34.9 (t, bridge), 34.1 ppm (t, bridge); due to weak intensity the carbonyl carbon atom was not detected. - **IR** (KBr): nu(tilde) = 2989 (w), 2950 (w), 2137 (vs), 1679 (vs), 1538 (w) 1245 (vs), 1189 (vs), 1127 (w), 998 (s), 823 $cm^{-1}$ (w). - **UV/Vis** ($CH_3CN$): $\lambda_{max}$ (lg ε) = 322 (3.33), 268 nm (4.29). - **MS** (EI, 70 eV): *m/z* (%) = 346 (1) [$M^+$], 290 (30), 263 (6), 145 (100), 131 (12), 116 (12),

90 (32), 77 (9), 44 (9). – **HRMS:** not available due to very low intensity of [$M^+$].

**4,16-Diisocyanato[2.2]paracyclophane (28):** The crude 4,16-bis(azidocarbonyl)[2.2]paracyclophane (**27**, 0.42 g, 1.21 mmol) was dissolved in anhydrous toluene (50 mL) under $N_2$ and the mixture was refluxed for 1 h. The solvent was evaporated *in vacuo* and the crude pale yellow residue of 0.34 g was subjected to column chromatography using a mixture of $CH_2Cl_2$ and pentane (1:4) on silica gel yielding 4,16-diisocyanato[2.2]paracyclophane (**28**, 0.10 g, 30%) as colorless crystalline solid. Recrystallization from $CH_2Cl_2$ and pentane by the diffusion method gave colorless plates suitable for X-ray analysis. – **m. p.**: 156 °C. – **$^1$H NMR** (400.1 MHz, $CDCl_3$): δ = 6.77 (dd, 2 H, $J_{7\text{-H},5\text{-H}}$ = $J_{15\text{-H},13\text{-H}}$ = 1.83 Hz, $J_{7\text{-H},8\text{-H}}$ = $J_{15\text{-H},16\text{-H}}$ = 7.91 Hz, 7-H, 15-H), 6.37 (d, 2 H, $J_{8\text{-H},7\text{-H}}$ = $J_{16\text{-H},15\text{-H}}$ = 7.91 Hz, 8-H, 16-H), 5.89 (d, 2 H, $J_{5\text{-H},7\text{-H}}$ = $J_{13\text{-H},15\text{-H}}$ = 1.74 Hz, 5-H, 13-H), 3.33–3.26 (m, 2 H, 2-H, 10-H), 3.04–2.92 (m, 4 H, 1-H, 9-H), 2.72–2.64 ppm (m, 2 H, 2-H, 10-H). – **$^{13}$C NMR** (100.6 MHz, $CDCl_3$): δ = 178.5 (s, C-18, C-21), 140.9 (s, C-6, C-14), 133.8 (s, C-4, C-12), 133.7 (d, C-8, C-16), 133.5 (s, C-3, C-11), 131.7 (d, C-5, C-13), 126.7 (d, C-7, C-15), 33.2 (t, C-1, C-9), 32.0 ppm (t, C-2, C-10). – **IR** (KBr): nu(tilde) = 2964 (w), 2272 (vs), 2253 (vs), 1559 (w), 867 (w), 720 (w), 628 $cm^{-1}$ (w). – **UV/Vis** ($CH_3CN$): $\lambda_{max}$ (lg ε) = 294 (2.97), 270 nm (3.26). – **MS** (EI, 70 eV): *m/z* (%) = 290 (25) [$M^+$], 258 (8), 256 (21), 160

(12), 145 (100), 128 (12), 90 (15), 71 (15), 57 (25), 44 (20). - **HRMS** ($C_{18}H_{14}N_2O_2$) calcd.: 290.105527, found: 290.10552 ± 3 ppm.

Table S1. Crystallographic data of **18**, **22**, **26**, and **28**.

| Compound | **18** | **22** | **26** | **28** |
|---|---|---|---|---|
| Formula | $C_{26}H_{32}N_2O_7$ | $C_{18}H_{20}N_2O$ | $C_{18}H_{20}Cl_2O_2$ | $C_{18}H_{14}N_2O_2$ |
| $M_r$ | 484.54 | 280.36 | 333.19 | 290.31 |
| Habit | colourless tablet | pale brown tablet | colourless tablet | colourless tablet |
| Cryst. size (mm) | 0.35 × 0.3 × 0.05 | 0.4 × 0.2 × 0.1 | 0.3 × 0.25 × 0.1 | 0.3 × 0.2 × 0.12 |
| Crystal system | orthorhombic | monoclinic | monoclinic | orthorhombic |
| Space group | $P2_12_12_1$ | $P2_1/c$ | $P2_1/c$ | $P2_12_12_1$ |
| Temperature (°C) | –140 | –140 | –140 | –140 |
| Cell constants: | | | | |
| *a* (Å) | 8.7908(12) | 13.158(2) | 8.4581(8) | 7.2803(8) |
| *b* (Å) | 10.6063(14) | 8.1464(14) | 11.5213(11) | 11.4247(12) |
| *c* (Å) | 25.502(4) | 14.985(3) | 7.5417(6) | 16.2522(16) |
| α (°) | 90 | 90 | 90 | 90 |
| β (°) | 90 | 116.039(6) | 99.890(4) | 90 |
| γ (°) | 90 | 90 | 90 | 90 |
| $V$ (Å$^3$) | 2377.7 | 1443.3 | 724.00 | 1351.8 |
| $Z$ | 4 | 4 | 2 | 4 |
| $D_x$ (Mg m$^{-3}$) | 1.354 | 1.290 | 1.528 | 1.426 |
| μ (mm$^{-1}$) | 0.10 | 0.08 | 0.45 | 0.10 |
| $F$(000) | 1032 | 600 | 344 | 608 |
| $2\theta_{max}$ | 60 | 60 | 60 | 60 |
| Refl. measured | 27503 | 16011 | 8191 | 10974 |
| Refl. indep. | 3922 | 4214 | 2119 | 2264 |
| $R_{int}$ | 0.041 | 0.049 | 0.031 | 0.041 |
| Parameters | 324 | 199 | 100 | 199 |
| Restraints | 1 | 0 | 0 | 0 |
| $wR(F^2$, all refl.) | 0.102 | 0.146 | 0.088 | 0.105 |
| $R(F, >4s(F))$ | 0.037 | 0.052 | 0.032 | 0.039 |
| $S$ | 1.05 | 1.01 | 1.05 | 1.03 |
| max. Δρ (e Å$^{-3}$) | 0.55 | 0.41 | 0.53 | 0.30 |

**References**


1. Sheldrick, G. M. *Acta Cryst*. **2008**, *A64*, 112–122.

2. Allgeier, H.; Siegel, M. G.; Helgeson, R. C.; Schmidt, E.; Cram, D. J. *J. Am. Chem. Soc*. **1975**, *97*, 3782-3789.

3. Antonov, D. Yu.; Sergeeva, E. V.; Vorontsov, E. V.; Rozenberg, V. I. *Russ. Chem. Bull*. **1977**, *46*, 1897-1900.

4. Reich, H. J.; Cram, D. J. *J. Am Chem. Soc*. **1969**, *91*, 3517-3526.

5. Yeh, Y. L.; Gorham, W. F. *J. Org. Chem.* **1969**, *34*, 2366-2370.
